# INI1-negative colorectal undifferentiated carcinoma with rhabdoid features and postoperative rapidly growing liver metastases: a case report and review of the literature

**DOI:** 10.1186/s40792-021-01189-5

**Published:** 2021-04-27

**Authors:** Masatsugu Kojima, Toru Miyake, Tomoyuki Ueki, Hiroyuki Ohta, Ryoji Kushima, Masanori Shiohara, Hiroo Mizuta, Hiroya Iida, Tsuyoshi Yamaguchi, Sachiko Kaida, Katsushi Takebayashi, Hiromitsu Maehira, Yusuke Nishina, Tomoharu Shimizu, Eiji Mekata, Masaji Tani

**Affiliations:** 1 Division of Gastrointestinal, Breast and General Surgery, Department of Surgery, Shiga University of Medical Science, Tsukinowa-cho, Seta, Otsu, Shiga 520-2192 Japan; 2 Department of Comprehensive Surgery, Shiga University of Medical Science, Tsukinowa-cho, Seta, Otsu, Shiga 520-2192 Japan; 3 Department of Pathology, Shiga University of Medical Science, Tsukinowa-cho, Seta, Otsu, Shiga 520-2192 Japan; 4 Department of Comprehensive Internal Medicine, Shiga University of Medical Science, Tsukinowa-cho, Seta, Otsu, Shiga 520-2192 Japan; 5 Medical Safety Section, Shiga University of Medical Science, Tsukinowa-cho, Seta, Otsu, Shiga 520-2192 Japan

**Keywords:** INI1/*SMARCB1*, Colorectal undifferentiated carcinoma, Rhabdoid feature, Rhabdoid tumor

## Abstract

**Background:**

Malignant tumors with rhabdoid features are extremely rare. They can occur in various organs, including the gastrointestinal tract, with common clinical features of high malignancy and poor prognosis.

**Case presentation:**

A 41-year-old man visited our hospital complaining of lower abdominal pain and fever. Computed tomography (CT) revealed two wall-thickening lesions in the rectum and sigmoid colon, with the latter invading the small intestine and abdominal wall. Lymph nodes were swollen in the sigmoid mesocolon and at the roots of the inferior mesenteric artery. Colonoscopy revealed a circular type 3 lesion in the sigmoid colon and a semicircular type 2 lesion in the rectum. Biopsies of the sigmoid colon and rectum lesions revealed poorly and moderately differentiated adenocarcinoma cells, respectively. The sigmoid colon, rectum, invaded small intestine, and abdominal wall were resected; lymph node dissection was also performed. Histopathological finding of the sigmoid colon lesion revealed that the tumor cells had poor connectivity with each other, and each cell had eosinophilic cytoplasm and a polymorphic nucleus. These characteristics are termed rhabdoid features, because the morphology of these cells is similar to that of rhabdomyosarcoma tumor cells. Immunohistochemical examination showed that the tumor cells were positive for both epithelial (cytokeratin AE1/AE3) and mesenchymal cell markers (vimentin); however, they were negative for integrase interactor 1 (INI1). Therefore, the sigmoid colorectal cancer was diagnosed as an INI1-negative undifferentiated carcinoma with rhabdoid features. The patient continued to experience high fever after surgery; thus, we performed an abdominal CT scan that revealed cystic lesions in the liver 4 days after surgery. These were absent in the positron emission tomography (PET)-CT scan performed 14 days before surgery. These tumors grew rapidly, and fine needle aspiration cytology revealed that they were undifferentiated carcinomas compatible with metastatic lesions from the undifferentiated carcinoma with rhabdoid features from the sigmoid colon. Chemotherapy was administered but was not effective. The patient died 60 days after surgery.

**Conclusions:**

INI1-negative colorectal undifferentiated carcinomas with rhabdoid features are extremely rare, have high histological malignancy, and a poor prognosis. Chemotherapy is not effective. Effective systemic therapy is desired.

## Background

Malignant tumors with rhabdoid features are extremely rare. They occur in various organs, including the gastrointestinal tract, and they have common clinical features such as high malignancy and poor prognosis [1]. Integrate interactor 1 (INI1) is a protein encoded by the tumor suppressor gene *SMARCB1*. INI1-deficient colorectal carcinoma has a high histological malignancy, large tumor diameter, and poor prognosis [2]. Approximately half of malignant tumors with rhabdoid features in the gastrointestinal tract lack INI1 expression [1]. Here, we report the case of an extremely rare INI1-negative undifferentiated colorectal carcinoma with rhabdoid features in the sigmoid colon, which caused the rapid progression of liver metastases after surgery.

## Case presentation

**Fig. 1:**
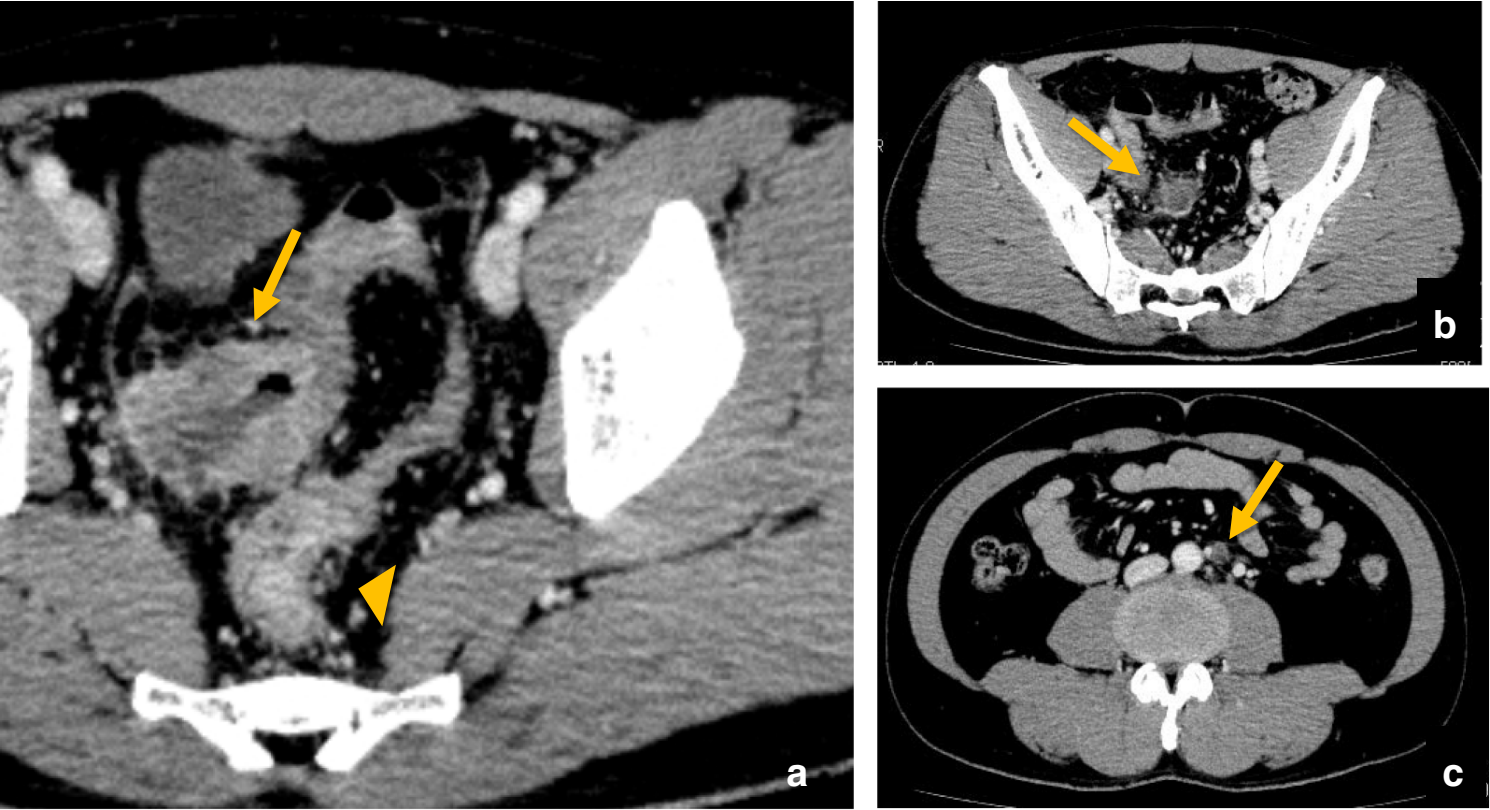
Computed tomography (CT) scan showed two wall-thickening lesions in the rectum (arrowhead) and sigmoid colon (arrow), with the latter invading the small intestine and abdominal wall (**a**). Lymph nodes were swollen in the sigmoid mesocolon (**b**) and at the roots of the inferior mesenteric artery (**c**). Some of the swollen lymph nodes had sac-like degeneration inside (**b**)

**Fig. 2:**
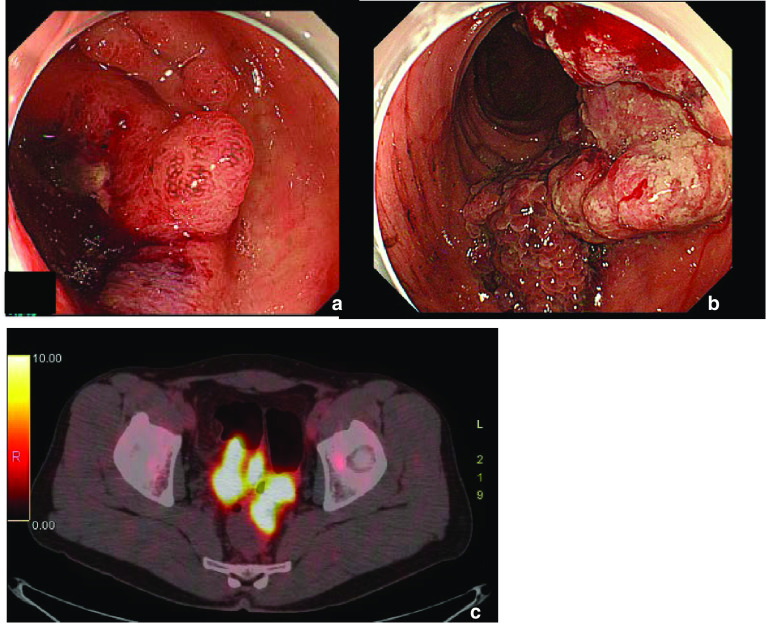
Colonoscopy revealed a circular type 3 lesion in the sigmoid colon (**a**) and a semicircular type 2 lesion in the rectum (**b**). A positron emission tomography (PET)-CT scan showed fluorodeoxyglucose (FDG) accumulation in the sigmoid colon lesion, rectal lesion, and swollen lymph nodes (**c**)

**Fig. 3:**
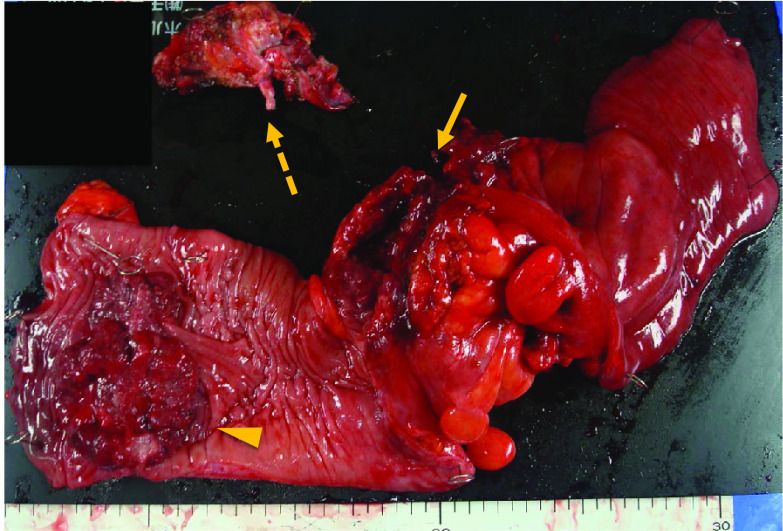
Macroscopic findings of the resected specimen. A circumferential type 3 lesion was found in the sigmoid colon (arrow), and a semicircular type 2 lesion was found in the rectum (arrowhead). The sigmoid colon lesion invaded the small intestine, vas deferens (dashed arrow), which were resected together

A 41-year-old man visited our hospital complaining of lower abdominal pain and fever for 2 weeks. He did not have a remarkable medical history. The detailed family history was unknown, because he and his parents were not close to their relatives. He had no siblings or children, and what they knew for sure was that his parents and grandparents had no history of cancer. Laboratory findings revealed mild inflammation; WBC was 8,940/μL, and CRP level was 1.08 mg/dL. He also had mild anemia with his hemoglobin level at 11.1 g/dL. The levels of the tumor markers, CEA and CA19-9 were within the normal range; moreover, sIL-2R levels were normal. The computed tomography (CT) scan showed two wall-thickening lesions in the rectum and sigmoid colon, with the latter invading the small intestine and abdominal wall (Fig. 1a). Lymph nodes were swollen in the sigmoid mesocolon and at the roots of the inferior mesenteric artery, which had sac-like degeneration inside (Fig. 1b, c). A colonoscopy revealed a circular type 3 lesion in the sigmoid colon and a semicircular type 2 lesion in the rectum (Fig. 2a, b). The biopsy result of rectum lesion was moderately differentiated adenocarcinoma; and the biopsy of the sigmoid colon lesion showed that round cells with swollen nuclei and eosinophilic cytoplasm proliferated separately with each other. We suspected that the sigmoid colon lesion was poorly differentiated adenocarcinoma. A positron emission tomography (PET)-CT scan showed fluorodeoxyglucose (FDG) accumulation in the sigmoid colon lesion, rectal lesion, and swollen lymph nodes; however, no distant metastases were demonstrated (Fig. 2c). We preoperatively diagnosed the case as rectal and sigmoid colon cancer with lymph node metastases without distant metastases. Sigmoidectomy, low anterior resection, and lymph node dissection were performed. The sigmoid colon cancer invaded into the small intestine, abdominal wall, and vas deferens, which were resected together. The tumor and sigmoid mesocolon were markedly swollen and occupied the pelvic entrance; moreover, the sigmoid mesocolon was strongly adhered to the retroperitoneum due to inflammation. White nodules that were present in the sigmoid mesocolon were removed; we suspected that these might be involved in peritoneal dissemination. The excised specimens are shown in Fig. 3.

**Fig. 4:**
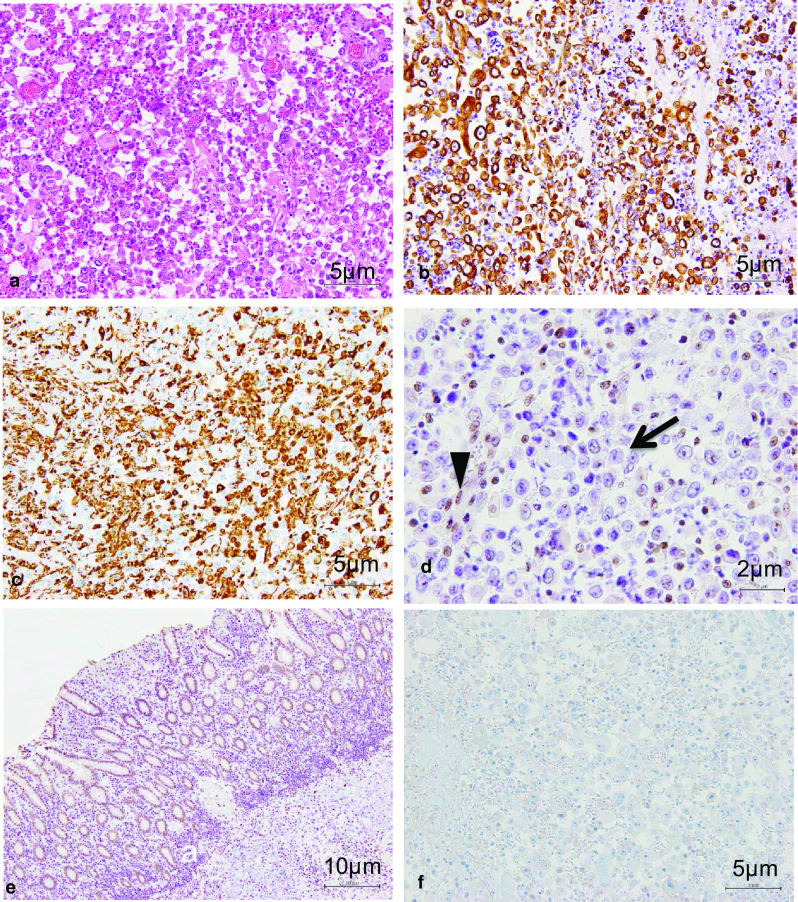
Sigmoid colon tumor cells had polymorphic nuclei and eosinophilic cytoplasm. These cells had poor connectivity with each other. These characteristics are called rhabdoid features, because the morphology of these cells is similar to that of rhabdomyosarcoma tumor cells (HE × 200) (**a**). Immunohistochemical examination showed that the tumor cells were positive for both epithelial (cytokeratin AE1/AE3) (× 200) (**b**) and mesenchymal cell markers (vimentin) (× 200) (**c**). These tumor cells were negative for INI1 (arrow), on the other hand, the surrounding non-neoplastic cells were positive for INI1 (arrowhead) (× 400) (**d**). Contrast to the tumor cells, non-tumor crypt epithelium cells were INI1 positive (× 100) (**e**). The tumor cells were negative for CDX2, which is a marker of colonocytes differentiation (× 200) (**f**)

Histopathological findings revealed that the rectal cancer was a typical moderately differentiated adenocarcinoma. However, the sigmoid colon cancer had different features including tumor cells with polymorphic nuclei and eosinophilic cytoplasm. These cells had poor connectivity with each other. These characteristics are called rhabdoid features, because the morphology of these cells is similar to that of rhabdomyosarcoma tumor cells (Fig. 4a). Immunohistochemical examination showed that the tumor cells were positive for both epithelial (cytokeratin AE1/AE3) and mesenchymal cell markers (vimentin). However, these cells were negative for INI1, which is normally expressed in the nucleus of cells (Fig. 4b–e). Therefore, the sigmoid colon cancer was diagnosed as an INI1-negative undifferentiated carcinoma with rhabdoid features. Moreover, CDX2, which is a marker of colonocytes differentiation, was negative (Fig. 4f); and Ki67, which shows proliferative ability, was as high as 40%. The white nodules on the sigmoid mesocolon were diagnosed as peritoneal dissemination, and the multiple lymph nodes were also diagnosed as metastatic. The final diagnosis was sigmoid colon cancer; pT4b (small intestine, abdominal wall) N2b M1c pStage IVC (peritoneal dissemination), and rectal cancer; pT3(SS) N0.

**Fig. 5:**
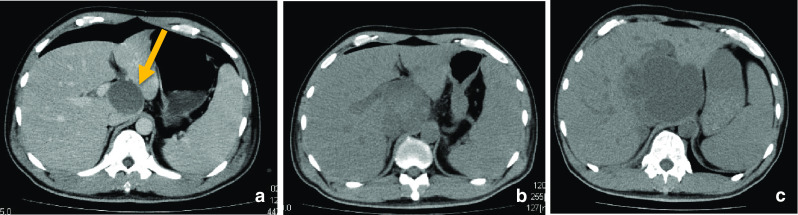
Abdominal CT revealed new cystic lesions appeared in liver segment S1 (**a**, arrow) and S7 (not shown) 4 days after surgery. These lesions grew rapidly 11 days after surgery (**b**). The fine needle aspiration cytology revealed that these were metastatic lesions from the undifferentiated carcinoma with rhabdoid features. Chemotherapy was ineffective and they continued to progress 47 days after surgery (**c**)

On day 4 after surgery, the patient continued to experience high fever of ≥ 38 °C. To examine the presence of lesions that could be causing the fever, an abdominal CT scan was performed, which revealed cystic lesions in liver segments S1 and S7 (Fig. 5a). These lesions were absent in the PET-CT scan performed 14 days before surgery. These lesions grew rapidly 11 days after surgery, as confirmed by CT (Fig. 5b); therefore, we suspected that the lesions were liver abscesses or metastases. A fine needle aspiration cytology revealed undifferentiated carcinoma cells compatible with metastatic lesions from the undifferentiated carcinoma with rhabdoid features from the sigmoid colon. Chemotherapy, comprising modified FOLFOX6 (oxaliplatin and an infusion of 5-fluorouracil/leucovorin (5FU/LV)) plus bevacizumab, was administered on postoperative day 22. However, after the second course, CT showed that the number and size of the liver metastases had progressed by postoperative day 47 (Fig. 5c). The molecular analysis indicated that the sigmoid colon cancer was KRAS and NRAS-wild, BRAFV600E-mutant, and microsatellite stable (MSS). The chemotherapy regimen was then changed to FOLFOXIRI (irinotecan, oxaliplatin, and an infusion of 5FU/LV) plus bevacizumab; however, the general condition of the patient rapidly deteriorated, and he died on postoperative day 60.

## Discussion

Malignant tumors with rhabdoid features are extremely rare and were first reported by Beckwith and Palmer in 1978 as a sarcomatoid subtype of Wilms tumor in the kidneys of fetuses and children [3]. Since then, it has been reported that malignant tumors with rhabdoid features can occur in various organs other than the kidneys in adults. Despite this diversity, these tumors have common clinical features, such as extremely high malignancy and poor prognosis [1, 2]. Malignant tumors with rhabdoid features are associated with carcinoma, sarcoma, and carcinosarcoma, depending on the tissue it develops in [4, 5]. In colorectal adenocarcinomas, they are named undifferentiated carcinomas with rhabdoid features.

Malignant tumors with rhabdoid features have cells with eosinophilic cytoplasm, oval or circular nuclei with prominent nucleoli, and vitreous inclusion bodies [6]. Although these tumors are morphologically sarcoma-like, immunohistochemical examination shows them to be negative for muscular markers and positive for epithelial (e.g., cytokeratin AE1/AE3) and mesenchymal cell markers (e.g., vimentin) [7]. Tumor cells have low adhesion, high mitotic potential, and are prone to infiltrate other organs. These histological features were also observed in the present case; the liver metastases discovered after surgery increased extremely rapidly and aggressively in both number and size. Furthermore, we detected cystic degeneration due to central necrosis, which was similar to liver abscesses. A preoperative CT scan revealed that some lymph node metastases showed cystic degeneration.

Malignant tumors with rhabdoid features are of two types: the first is entirely composed of undifferentiated carcinoma cells (pure type), whereas the second consists of not only undifferentiated carcinoma cells but also carcinoma cells with a high degree of differentiation in developing organs (composite type). The presence of composite type suggests that the rhabdoid features could have been developed from dedifferentiating from differentiated malignant cells [1, 8].

INI1 is a protein encoded by the tumor suppressor gene, *SMARCB1*. It constitutes the switch/sucrose non-fermentable (SWI/SNF) complex, which is thought to function in an ATP-dependent manner to cause a conformational change in the nucleosome that alters histone-DNA binding, thereby facilitating transcription factor access [9–11]. INI1 is normally expressed in the nucleus of cells; however, its expression is known to be lack or reduced in rhabdoid tumor cells [9, 10]. In a study that examined 3051 cases of colorectal cancers, the INI1-encoding gene was deleted in 14 cases (0.46%) [2]. INI1-deficient colorectal carcinomas have a high histological malignancy, large tumor diameter, and poor prognosis [2]. Approximately half of gastrointestinal undifferentiated carcinomas with rhabdoid features lack INI1 expression [1]; INI1-encoding gene deletion appears to be crucial for the development of rhabdoid features in some cases [1, 10, 11].

Large intestine-derived malignant tumors with rhabdoid features are rare [12], and to date, only 28 cases (including the current case) of colorectal undifferentiated carcinoma with rhabdoid features have been reported (Table 1) [1, 5, 12–36]. These cases have been reported in 17 men and 11 women. The average age of these patients was 64.5 years (range: 23–87 years). The right colon (cecum to transverse colon) was the most common site of carcinoma occurrence in 16 cases (57.1%), followed by the left colon (descending colon to sigmoid colon) in 6 cases (21.4%), and the rectum in 6 cases (21.4%). The average tumor diameter was 9.4 cm (range, 4–17 cm). Of all, the lymph nodes metastases were reported in 20 cases (71.4%), the liver in 7 cases (25%), peritoneal dissemination in 4 cases (14.3%), and the lungs in 2 cases (7.1%). Regarding INI1, 57% reported cases were negative, which was almost the same rate that reported before in gastrointestinal tract undifferentiated cancer with rhabdoid features [1].

**Table 1:** Reported colorectal cancer with rhabdoid features (*N* = 28)

No	Author	Year	Age	Gender	Site	Size (cm)	Treatment	Histology	Outcome	Metastasis	INI1 expression	RAS, BRAF, MSI
1	Chetty et al. [13]	1993	72	M	Cecum	6 × 5x2	Surgery	Composite	Dead 3 mo	Liver, LN	ND	ND
2	Yang et al. [14]	1994	75	M	Transverse	15 × 10x5	Surgery	Pure	Dead 0.5 mo	LN	ND	ND
3	Macak et al. [15]	1995	50	M	Transverse	6 × 5x3.5	Surgery	Composite	Not reported	LN	ND	ND
4	Marcus et al. [16]	1996	84	F	Transverse	7	Surgery	Pure	Dead 3 mo	None	ND	KRAS, NRAS, HRAS WT
5	Nakamura et al. [17]	1999	76	M	Cecum	14 × 8x6	Surgery	Pure	Dead 3 mo	Liver, LN	ND	ND
6	Horie et al. [18]	2000	76	M	Rectum	NS	NS	Composite	NS	Liver	ND	ND
7	Hamada et al. [19]	2004	65	F	Rectum	13 × 8	Surgery	Pure	Alive 10 mo	Lung	ND	ND
8	Nakayama et al. [20]	2005	63	M	Rectum	10 × 8.4	Surgery	Pure	Alive 12 mo	LN	ND	ND
9	Matsunuma et al. [21]	2005	54	M	Ascending	11 × 9.5	Surgery	Pure	Dead 2 mo	None	ND	ND
10	Yamaguchi et al. [22]	2006	72	F	Descending	10 × 8.5	Surgery,CT	Composite	Dead 1.5 mo	Peritoneum, nodes	ND	ND
11	Kono et al. [5]	2007	66	M	Cecum	13 × 13	Surgery	Composite	Dead 1.5 mo	LN	ND	KRAS WT, MSS
12	Mastoraki et al. [23]	2009	62	F	Descending	8 × 10x8	Surgery	Pure	Dead 4 mo	Liver	ND	RAS family WT
13	Han et al. [24]	2010	23	F	Rectum	6 × 5	Surgery,CT,RT	Pure	Alive 17 mo	None	ND	ND
14	Pancione et al. [25]	2011	71	F	Asecnding	10 × 10	Surgery,CT	Pure	Dead 8 mo	Liver, peritoneum, LN	ND	BRAFV600E
15	Takeshita et al. [26]	2011	75	M	Rectum	17 × 10	Surgery,CT	Pure	Dead 5 mo	LN	ND	ND
16	Remo et al. [27]	2012	73	F	Ascending	10 × 4	Surgery,CT	Composite	Dead 6 mo	LN	negative	BRAF600VE
17	Kobayashi et al. [28]	2012	58	F	Ascending	10 × 5	Surgery	Composite	Dead 4 mo	LN	positive	ND
18	Lee JR et al. [29]	2013	62	M	Sigmoid	4 × 4x1	Surgery,CT	Composite	Alive 36 mo	LN	ND	ND
19	Samalavicius et al. [30]	2013	49	M	Rectum	7	Surgery,CT	Pure	Dead 7 mo	LN	positive	KRAS WT, BRAF600VE
20	Saito et al. [31]	2014	81	M	Cecum	8 × 6	Surgery,CT	Composite	Dead 0.7 mo	Liver, LN	ND	ND
21	Baba et al. [32]	2014	45	F	Sigmoid	9 × 8x5	Surgery	Composite	Dead 1.5 mo	Peritoneum, lung, LN	ND	ND
22	Romera et al. [33]	2014	77	M	Descending	-	Surgery	Pure	Dead 2 mo	None	ND	ND
23	Agaimy et al. [1]	2014	79	M	Cecum	9 × 5x2	Surgery	Pure	Dead 6 mo	LN	negative	BRAFV600E
24	Cho et al. [12]	2015	73	M	Cecum	4 × 3	Surgery	Composite	Alive 1 mo	LN	ND	KRAS WT, BRAF600E
25	Moussaly et al. [34]	2015	87	F	Transverse	12 × 9.5x8.5	Surgery	Composite	Dead 2 mo	None	ND	ND
26	Kalyan et al. [35]	2015	31	F	Cecum	7	Surgery,CT	Composite	Dead 4 mo	LN	positive	KRAS mutant, BRAF WT
27	D'Amico et al. [36]	2019	65	M	Ascending	10	Surgery	Pure	Alive 48 mo	LN	negative	ND
28	This study	2019	41	M	Sigmoid	7 × 6	Surgery,CT	Pure	Dead 2 mo	Liver, peritoneum, LN	negative	RAS WT, BRAFV600E, MSS

*CT* chemotherapy, *RT* radiotherapy, *mo* month, *LN* lymph node, *NS* not specified, *ND* not done, *WT* wild type, *MSI* microsatellite instability, *MSS* micorosatellite stable

Raf is a kinase that is a member of the Ras/Raf/MAPK pathway, and when this pathway is activated, it enhances the cell cycle. In BRAF, which is a member of the RAF family, a missense mutation (V600E) of exon 15 codon 600 activates the downstream signal [37]. About 7% of colorectal cancers have this mutation, and their prognosis are known to be poor [38]. In addition, such BRAF mutant colorectal cancer was reported to be more common in sporadic microsatellite instability (MSI) -high right-sided colon cancers [39]. Regarding undifferentiated colorectal cancer with Rhabdoid features, BRAF V600VE mutation was observed in 6 of 7 reported cases (Table 1). We supposed that there may be some correlation between BRAF mutation and rhabdoid features in colorectal cancers. The elucidation of the molecular mechanism is considered to be an issue for the future. On the other hand, MSI was examined in only 2 cases including our case. Both cases were microsatellite stable, but the number of cases was too small to make any conclusion. Along with the accumulation of cases, elucidation of the correlation between MSI status and the rhabdoid feature is also a future issue.

**Fig. 6:**
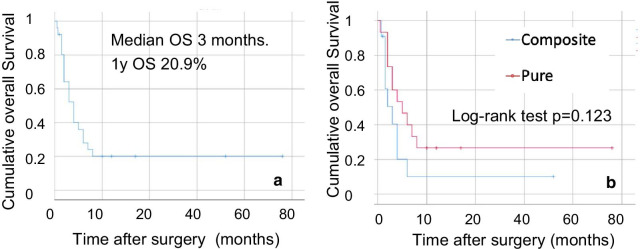
Kaplan–Meier survival analyses. (**a**) Overall survival (OS) of all patients with colorectal undifferentiated carcinoma with rhabdoid features was 3 months after surgery. The 1-year survival rate was 20.9%. (**b**) Comparison of patients with the pure and composite types showed no significant difference (*p* = 0.123)

The colorectal undifferentiated carcinoma with rhabdoid features reported were divided into 13 and 15 cases of the composite and pure types, respectively. The survival curve for these cases is shown in Fig. 6. The median overall survival (OS) of patients with undifferentiated carcinoma with rhabdoid features was 3 months, indicating an extremely poor prognosis. Comparison of the OS of patients with the pure and composite types showed no significant difference (*p* = 0.123). Although there is a limitation that various clinical stages are included in each type group, it suggests that the presence of rhabdoid features would be responsible for the poor survival [1]. The pure type might simply be a case in which the original cancer lesion could not be found in specimens, because it was too small. Although chemotherapy was administered to 9 patients with metastasis, 8 patients died within 1 year. This suggests that the effect of chemotherapy is limited. It has been reported at the experimental level in vitro and in vivo that gefitinib, a selective tyrosine kinase receptor inhibitor, is effective for treating malignant tumors with rhabdoid features [40]. It is considered that further effective clinical trials for malignant tumors with rhabdoid features is required.

## Conclusion

INI1-negative colorectal undifferentiated carcinomas with rhabdoid features are extremely rare, and they have aggressive histological malignancy and poor survival. Chemotherapy is not effective at present; therefore, future clinical trials is required to improve the survival.

## Data Availability

The data sets supporting the conclusions of this article are included within the article.

## Abbreviations

CT: Computed tomography
WBC: White blood cell
CRP: C-reactive protein
CEA: Carcinoembryonic antigen
CA19-9: Carbohydrate antigen 19-9
sIL-2R: Soluble interleukin-2 receptor
INI1: Integrate interactor 1
PET: Positron emission tomography
FDG: Fluorodeoxyglucose
5FU/LV: 5-Fluorouracil/leucovorin
